# Elevated Growth Temperature Modifies Drought and Shade Responses of *Fagus sylvatica* Seedlings by Altering Growth, Gas Exchange, Water Relations, and Xylem Function

**DOI:** 10.3390/plants14101525

**Published:** 2025-05-19

**Authors:** Faustino Rubio, Ismael Aranda, Rosana López, Francisco Javier Cano

**Affiliations:** 1 Departamento de Sistemas y Recursos Naturales, Escuela Técnica Superior de Ingeniería de Montes, Forestal y del Medio Natural, Universidad Politécnica de Madrid, 28040 Madrid, Spain; rosana.lopez@upm.es; 2 Instituto de Ciencias Forestales (ICIFOR-INIA), Consejo Superior de Investigaciones Científicas (CSIC), 28040 Madrid, Spain; aranda@inia.csic.es; 3 Hawkesbury Institute for the Environment, Western Sydney University, Richmond, NSW 2751, Australia

**Keywords:** drought, high temperature, shade, wood anatomy, hydraulic traits, biomass allocation, water relations, osmotic adjustment, leaf gas exchange, phenotypic plasticity

## Abstract

Climate change is increasing global temperatures and imposing new constraints on tree regeneration, especially in late-successional species exposed to simultaneous drought and low-light conditions. To disentangle the effects of warming from those of atmospheric drought, we conducted a multifactorial growth chamber experiment on *Fagus sylvatica* seedlings, manipulating temperature (25 °C and +7.5 °C above optimum), soil moisture (well-watered vs. water-stressed), and light intensity (high vs. low), while maintaining constant vapor pressure deficit (VPD). We assessed growth, biomass allocation, leaf gas exchange, water relations, and xylem hydraulic traits. Warming significantly reduced total biomass, leaf area, and water-use efficiency, while increasing transpiration and residual conductance, especially under high light. Under combined warming and drought, seedlings exhibited impaired osmotic adjustment, reduced leaf safety margins, and diminished hydraulic performance. Unexpectedly, warming under shade promoted a resource-acquisitive growth strategy through the production of low-cost leaves. These results demonstrate that elevated temperature, even in the absence of increased VPD, can compromise drought tolerance in beech seedlings and shift their ecological strategies depending on light availability. The findings underscore the need to consider multiple, interacting stressors when evaluating tree regeneration under future climate conditions.

## 1. Introduction

The significant increase in temperature due to ongoing climate change, along with more frequent and severe droughts (e.g., scenario SSP5-8.5) [1], poses challenges for forest species with long reproductive cycles, slow migration capacity, and largely unknown limits of acclimation. Plant acclimation to warmer growing temperatures remains an underexplored process and becomes increasingly complex when combined with other environmental stressors. Many plant species experience a triple interaction of stressors: sustained warming, episodic water deficit, and regeneration under the low-light conditions typical of forest understories [2]. To gain insights into how future warming will affect regeneration niches of key late-successional species, and how tree populations may respond to rapid environmental changes, it is essential to examine the acclimation capacity and the phenotypic plasticity, i.e., the capacity of an organism to modify its physiological, morphological, or developmental traits in response to environmental changes, compared to its baseline behavior under control conditions, of essential functional traits [3,4]. Shifts in traits such as gas exchange, water relations, and biomass allocation can influence ecological strategies like drought and shade tolerance, ultimately affecting the species’ ability to compete and regenerate, thereby altering forest dynamics [5,6].

The temperature dependence of most biological processes, such as metabolic and growth, follows an asymmetric Arrhenius equation, typically peaking at the optimal temperature [7]. For instance, warming can promote tree growth and increase leaf area at the expense of root investment, as long as water and nutrients are not limiting. However, growth tends to decline when temperatures exceed the optimum [8,9], mainly due to a reduction in leaf area and net photosynthesis (*A*_n_) [8,10,11]. Meanwhile, respiration rates (*R*_d_) continue to rise beyond the optimum for *A*_n_, leading to further declines in growth [12]. Reduced carbon availability at higher temperatures is also reflected in lower leaf mass per area (*LMA*) [13], a trait associated with plant ecological strategies, including drought and shade tolerance [14]. Additionally, leaves that develop under elevated temperatures tend to be smaller, which helps reduce the boundary layer and facilitate heat dissipation [15,16]. Both *A*_n_ and *R*_d_ acclimate to changes in growth temperature typically by the downregulation of *R*_d_ [12,17] and the upregulation of the temperature optimum for *A*_n_ [9,17,18,19], which allows plants to maximize carbon gain with warming [19]. However, a decoupling between *A*_n_ and stomatal conductance (*g*_sw_) has been reported at high temperatures in many species, particularly those adapted to warm climates. In such cases, *A*_n_ decreases while *g*_sw_ increases due to the so-called evaporative cooling [20,21,22], with the concomitant reduction in intrinsic water use efficiency (*iWUE*) and increased risk of dehydration [23]. 

The extent to which stomata acclimate to warming depends on a complex balance among their key roles. Stomata regulate not only photosynthesis and carbon uptake but also leaf water loss through transpiration (*E*), leaf temperature via evaporative cooling [20,21], responses to oxidative stress [24], and leaf water balance [25]. Maintaining water potential within specific safety limits is crucial to prevent hydraulic failure [26] and leaf dehydration [27]. Transpiration is driven by the vapor pressure difference between the leaf interior and the atmosphere (VPD*_L_*) multiplied by the total conductance for water vapor. Therefore, for a given stomatal aperture, more water is lost to the atmosphere as air temperature increases, due to higher VPD*_L_* [22,28]. To sustain higher water flow at higher evaporative demand, leaf water potential at the turgor loss point (*π*_0_) often decreases in seedlings grown at warmer temperatures [29], an adjustment frequently observed in species adapted to warm and dry habitats [30]. However, it remains unclear whether these changes are a direct response to warming alone or to its interaction with other stressors, such as soil or atmospheric moisture [31].

Acclimation of the hydraulic architecture to warming in forest species remains understudied even though changes are expected due to increased evaporative demand [9,29]. For example, well-watered *Populus tremuloides* seedlings grown at warmer temperatures than ambient showed higher growth, and greater allocation to roots, which increased hydraulic conductance and a higher sapwood area to leaf area (Huber value, *H_V_*) [29]. Conversely, in a xeric site, both *Juniperus monosperma* and *Pinus edulis* showed a lack of acclimation of *H_V_* or xylem anatomy after four years of atmospheric warming and only the juniper reduced leaf-specific hydraulic conductivity (*K*_L_), associated with a decrease in canopy conductance under warming [28]. Well-watered plants exposed to warming can develop larger and/or more frequent xylem conduits [29,32], potentially enhancing conductivity, without necessarily affecting embolism resistance. By contrast, in *Eucalyptus camaldulensis* and *E. grandis*, seedlings grown above optimal growth temperatures developed smaller vessels, lower specific conductivity (*K*_s_), and higher wood density (ρ) [33,34]. Besides changes in xylem anatomy, increased sap temperature reduces viscosity while maintaining constant sap density, thereby enhancing whole-plant hydraulic conductance independently of xylem structural traits [33].

Although warming increases the water deficit experienced by plants, the water deficit itself also amplifies the negative effects of supraoptimal growth temperatures [35,36]. Water deficit reduces the temperature optima for both photosynthesis, as a consequence of reduced diffusional CO_2_ conductance [37,38], and tree growth due to decreased photosynthesis rates, reduced leaf area, and increased *LMA* [8]. However, active osmotic adjustment in warmed trees can enhance drought tolerance [39]. Water deficit-induced stomatal closure increases leaf temperature and the risk of tissue damage, even when thermal acclimation occurs [15,40]. When stomata are partially or fully closed due to soil water deficit, residual water loss through the leaf cuticle becomes crucial for plant survival [41]. This leaf minimum conductance (*g*_min_) typically decreases under drought; however, short-term high temperatures can increase *g*_min_ [42], whereas prolonged exposure to elevated temperatures under sustained higher VPD tends to reduce it [23].

Another major environmental stressor limiting the growth and regeneration capacity of many temperate tree species is light availability [43]. Under shaded conditions, plants allocate more resources to stem elongation and increased leaf surface area to capture light [43], leading to trade-offs that can compromise acclimation to water stress [44,45]. For example, plants grown under shade often exhibit a lower capacity of osmotic adjustment [46,47], lower *LMA* [13], and lower biomass allocation to roots [48], which collectively impair drought and heat tolerance. Conversely, large forest gaps expose seedlings to high light intensity and warmer temperatures, promoting higher metabolic activity and growth rates but also exposing them to greater water stress and thermal damage [15,49]. Understanding how elevated temperatures, water stress, and light conditions interact is critical for predicting challenges to forest regeneration under climate change since the combined response to multiple stressors is not simply the additive sum of individual stress responses [50,51].

Most studies on temperature acclimation have not controlled for air humidity, resulting in elevated VPD that can confound the interpretation of warming effects, given the high sensitivity of transpiration to VPD [29]. Furthermore, transpiration responses are known to acclimate to the prevailing VPD during growth [52], making comparisons across studies conducted at different VPD levels potentially controversial [53]. By contrast, studies that directly manipulate temperature while maintaining constant VPD are rare [54]. Disentangling the direct effects of warming from soil and atmospheric drought thus requires further investigation, as shifts in resource allocation, along with morphological and physiological acclimation to warming, can increase vulnerability to subsequent drought events [9,55].

In this study, we exposed one-year-old seedlings of *Fagus sylvatica* L. to a factorial combination of optimal (25 °C; T25) and supraoptimal (+7.5 °C; T32) temperatures, soil water availability (well-watered vs. water-deficit), and light intensity (high vs. low), while maintaining a constant VPD. Our goal was to disentangle the direct effects of elevated temperature from atmospheric drought and to investigate how warming modulates the physiological, morphological, and anatomical responses of beech seedlings to simultaneous water and light stress (see Table A1 for measured traits and abbreviations). By addressing these questions, our study provides novel insights into the multifactorial stress responses of a key late-successional species and advances our understanding of its regeneration potential under projected climate change scenarios [51,56]. We hypothesized that: (1) Warming will reduce plant growth, primarily through a reduction in leaf area rather than proportional declines in photosynthesis, exacerbating the negative effects of concurrent water deficits and shade. (2) Stomatal conductance will increase under warming when water is not limiting to promote evaporative cooling, but it will decrease under water deficits. (3) Xylem anatomical traits related to hydraulic conductivity will adjust to match changes in leaf transpiration, maintaining water balance. (4) Phenotypic plasticity to water and light availability will be reduced due to warming.

## 2. Results

### 2.1. Changes in Morphology and Biomass Allocation

**Figure 1:**
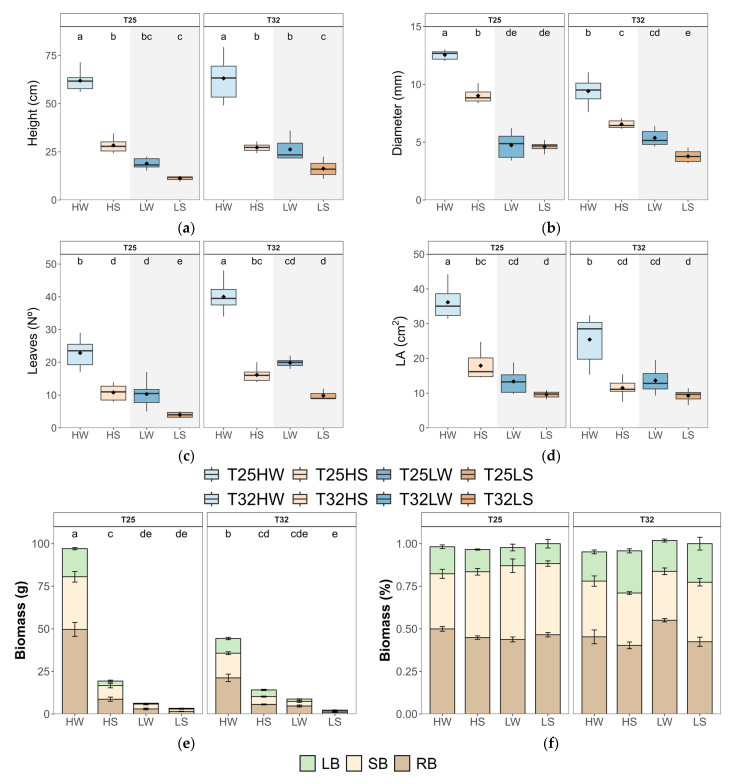
Boxplots of growth and biomass allocation of beech seedlings grown in a factorial combination of temperature: 25 °C (T25) vs. 32 °C (T32), soil water availability: well-watered (W) vs. water-stressed (S), and light intensity: high (H) vs. low (L). (**a**) height (*h*); (**b**) diameter (*d*); (**c**) a number of developed leaves (*leaves*); (**d**) leaf area (*LA*); (**e**) total biomass of leaves (*LB*), shoots (*SB*), and roots (*RB*) and (**f**) relative proportion of biomass (%) of leaves (*LB*), shoots (*SB*), and roots (*RB*). The straight horizontal black line within each boxplot indicates the median, while the mean value is represented as a solid black point. Different letters indicate significant differences among treatments.

Elevated growth temperature (T32) significantly reduced overall plant growth and altered biomass allocation in *Fagus sylvatica* seedlings compared to optimal conditions (T25). After 90 days, seedlings grown at T25 developed thicker stems and larger leaves, and accumulated more total biomass (Figure 1; Table A2). In contrast, those grown at T32 produced a greater number of smaller leaves, leading to lower total leaf area (*TLA*) despite an increase in leaf number (Figure 1). This pattern was consistent across all water and light treatments, indicating a direct effect of supraoptimal temperature on growth morphology. Temperature also reshaped biomass partitioning. Seedlings at T32 invested proportionally more in leaves and less in shoots than those at T25. The shift toward greater leaf allocation may reflect a compensatory response to reduced leaf size, but it did not offset the reduction in carbon gain. Growth at T32 was tightly coupled with lower net photosynthesis (*A_n_*), as confirmed by strong correlations between stem diameter and *A_n_* across treatments (Figure A6). Both water deficit and low light reduced growth, biomass, and the number of fully developed leaves, although the cumulative effect of light intensity exerted a stronger effect than water deficit (Figure 1). Water deficit enhanced root biomass allocation in both temperature regimes and increased water use efficiency, while shade decreased overall plant biomass (Figure 1). However, under all stress combinations, seedlings at T32 showed lower growth performance and reduced plasticity in allocation patterns compared to T25 (Table A2; Figure A2). These results demonstrate that warming directly constrains growth through reduced carbon assimilation and altered morphology, irrespective of resource availability.

### 2.2. Leaf Gas Exchange, Water Use Efficiency, and Stomatal Traits

**Figure 2:**
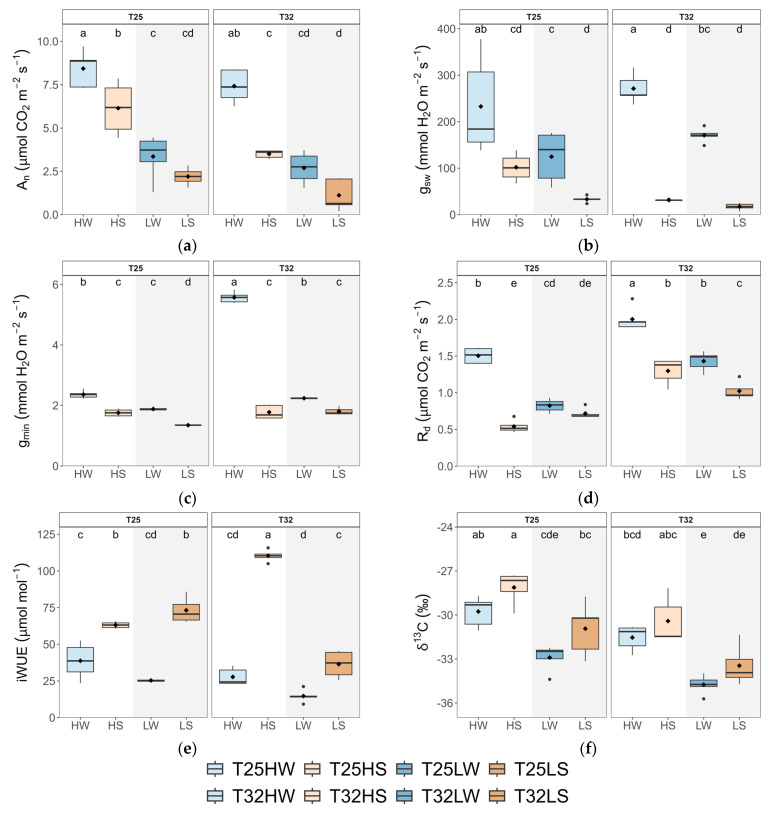
Boxplots of leaf gas exchange and isotopic carbon composition of beech seedlings grown in a factorial combination of temperature: 25 °C (T25) vs. 32 °C (T32), soil water availability: well-watered (W) vs. water-stressed (S), and light intensity: high (H) vs. low (L): (**a**) net assimilation (*A*_n_); (**b**) stomatal conductance to water vapor (*g*_sw_); (**c**) minimal conductance (*g*_min_); (**d**) mitochondrial respiration (R_d_); (**e**) intrinsic water use efficiency (*iWUE*); (**f**) carbon isotope composition (*δ^13^C*). The straight horizontal black line within each boxplot indicates the median, while the mean value is represented as a solid black point. Different letters indicate significant differences among treatments.

Leaf gas exchange was significantly affected by temperature, with T32 reducing photosynthetic performance and water-use efficiency compared to T25. Seedlings at T32 exhibited significantly lower *A*_n_ and higher mitochondrial respiration (*R*_d_), which together reduced the net carbon balance across all treatments (Figure 2; Table A2). Notably, this decline in *A*_n_ occurred even though VPD was held constant across temperature regimes, highlighting a direct temperature effect independent of evaporative demand. Interestingly, stomatal conductance (*g*_sw_) increased under T32, but only in well-watered conditions, suggesting that stomata remained more open to enhance evaporative cooling at higher leaf temperatures. This increase in *g*_sw_, however, was not matched by higher *A*_n_, leading to significantly lower intrinsic water use efficiency (*iWUE*) at T32. The more negative *δ^13^C* values observed under T32 further confirmed the decreased water-use efficiency (Figure 2e,f). Under water stress, *g*_sw_ decreased across both temperature regimes, but the reduction in gas exchange was more pronounced at T32, indicating a compounding effect of warming and drought (Table A2). Temperature also modulated residual water loss. Minimum leaf conductance (*g*_min_) was significantly higher at T32, particularly in well-watered plants, suggesting greater cuticular or incomplete stomatal closure under warm conditions. Stomatal traits were not directly affected by warming although warming had significant interaction with the other two growing factors. Stomatal density (*S*_D_) increased with light intensity only at T25, but stomatal length (*L*_s_) was shorter under water deficit only at T32.

### 2.3. Leaf Water Relations

**Figure 3:**
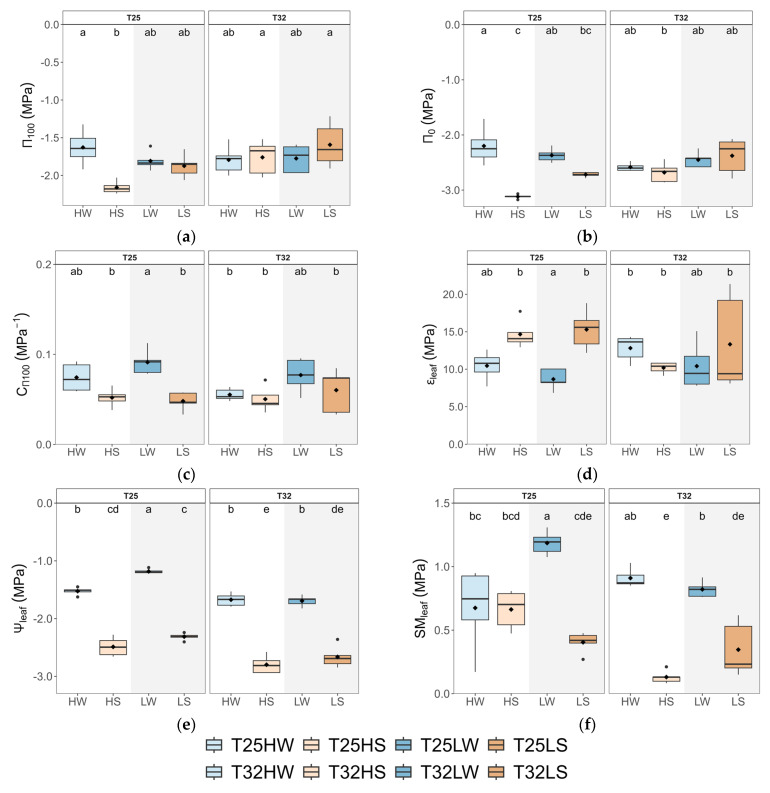
Boxplots of leaf water relations of beech seedlings grown in a factorial combination of temperature: 25 °C (T25) vs. 32 °C (T32), soil water availability: well-watered (W) vs. water-stressed (S), and light intensity: high (H) vs. low (L): (**a**) osmotic potential at full turgor (*π*_100_); (**b**) osmotic potential at turgor loss point (*π*_0_); (**c**) leaf capacitance before the turgor loss point (*C_π_*_100_); (**d**) maximum modulus of elasticity (*ε_leaf_*); (**e**) leaf midday water potential (*Ψ_leaf_*); and (**f**) safety margin (*SM_leaf_*). The straight horizontal black line within each boxplot indicates the median, while the mean value is represented as a solid black point. Different letters indicate significant differences among treatments.

Elevated temperature (T32) negatively affected leaf water status, primarily by lowering midday leaf water potential (*Ψ_leaf_*), stem water potential (*Ψ_stem_*), and predawn water potential (*Ψ_pd_*), regardless of water or light treatment (Figure 3; Table A2). These reductions were most pronounced under water deficit, but even well-watered plants at T32 exhibited more negative water potentials compared to those at T25, pointing to a direct thermal effect on plant water balance. The capacity for osmotic adjustment was also compromised at T32. At T25, seedlings under water stress showed clear osmotic adjustments, with more negative osmotic potential at full turgor (*π*_100_) and turgor loss point (*π*_0_), especially under high light (Figure 3). These responses were accompanied by higher leaf capacitance (*C_π_*_100_) and lower leaf tissue elasticity (*ε_leaf_*), allowing for greater water retention and stress buffering. However, at T32, seedlings failed to significantly adjust π_0_ or *π*_100_ under stress, particularly under high light, suggesting a loss of drought acclimation capacity. Notably, warming also reduced the leaf safety margin (*SM_leaf_*)—the difference between midday *Ψ_leaf_* and *π*_0_—across all treatments. This decrease was the most severe under combined water deficit and shade, where *SM_leaf_* values approached critical thresholds, increasing the risk of turgor loss and hydraulic failure.

### 2.4. Hydraulic Traits and Stem Anatomy

**Figure 4:**
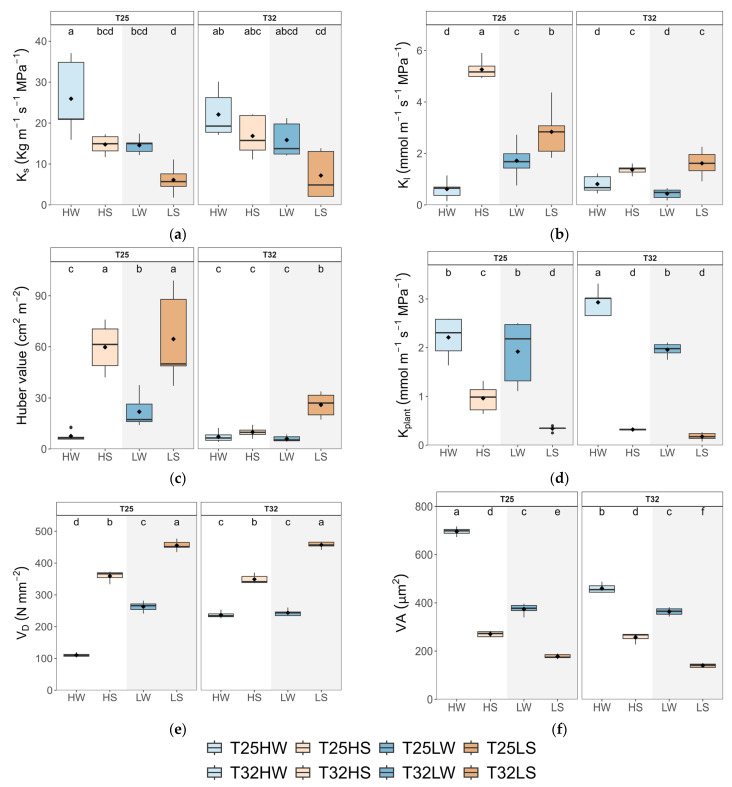
Boxplots of hydraulic traits and stem anatomy of beech seedlings grown in a factorial combination of temperature: 25 °C (T25) vs. 32 °C (T32), soil water availability: well-watered (W) vs. water-stressed (S), and light intensity: high (H) vs. low (L): (**a**) stem specific hydraulic conductivity (*K*_s_); (**b**) leaf-specific hydraulic conductivity (*K*_leaf_); (**c**) Huber value (*H*_v_); (**d**) plant hydraulic conductance (*k*_plant_); (**e**) vessel density (*V*_D_); and (**f**) average vessel area (*V*_A_). The straight horizontal black line within each boxplot indicates the median, while the mean value is represented as a solid black point. Different letters indicate significant differences among treatments.

Elevated temperature (T32) significantly affected several key hydraulic traits, particularly in interaction with light and water availability (Table A2). While stem-specific hydraulic conductivity (*K_s_*) was primarily influenced by light intensity, leaf-specific conductivity (*K_L_*) and the Huber value (*H*_v_) showed consistent reductions under warming, especially under water stress or shade (Figure 4, Table A2). Notably, plant hydraulic conductance (*k*_plant_) increased slightly only under high light and well-watered conditions at T32, suggesting a limited compensatory response to warming when resources were not limiting. Contrary to expectations, warming alone did not significantly alter *K*_s_, but it reduced *K*_L_ and *H*_v_ in most treatments (Figure 4), indicating a decoupling between stem-level and whole-plant hydraulic efficiency. This effect was particularly evident in shaded or drought plants, where warming exacerbated the reduction in water transport efficiency. The mean loss of xylem conductivity (*PLC*) was lower than 10% in well-watered plants and lower than 16% in water-stressed plants pointing to moderate drought during the whole experiment.

Stem xylem anatomy was remarkably affected by water stress, which induced an increase in vessel density (*V*_D_) but lowered vessel area (*V*_A_) and hydraulic diameter (*D*_h_). High light favored wider vessels (higher *V*_A_) and lower vessel density (*V*_D_), which were positively correlated with total leaf area (*TLA*) across treatments (Figure A7). Importantly, warming constrained this anatomical plasticity by limiting the increase in *V*_A_ typically observed under high light. No significant differences between treatments were observed in the xylem area occupied by radial parenchyma.

### 2.5. Plasticity in Response to Temperature, Light, and Water Availability

**Figure 5:**
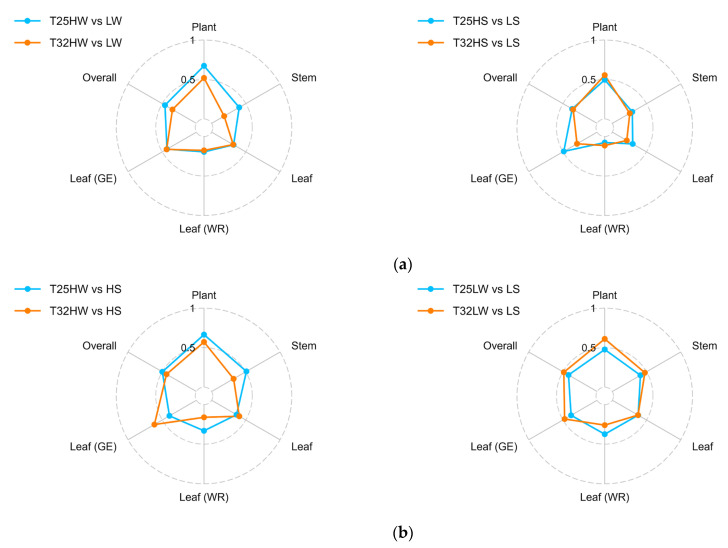
Radar plots of temperature influence over the Phenotypic Plasticity Index (*PPi*) in response to different stressors: (**a**) light intensity and (**b**) water availability. Traits were grouped into different organizational levels according to Table A1 into Plant, Stem, Leaf (including both Water relations + Gas exchange), Leaf (WR) (leaf water relations only), Leaf (GE) (leaf gas exchange only), and overall. Treatments are as follows: growth temperatures (T25, T32); HW (high light intensity and well-watered), HS (high light intensity and water stress), LW (shade and well-watered), LS (shade and water stress).

Warming (T32) consistently reduced the plasticity of many traits (quantified by the phenotypic plasticity index, *PPi*), especially those related to hydraulics, growth, and leaf water relations, indicating a dampened capacity to adjust to environmental variation under elevated temperature (Figure 5). Under well-watered conditions, *PPi* in response to light was higher at T25 for most traits related to growth, gas exchange, and biomass allocation. Under water stress, T32 seedlings exhibited reduced plasticity in leaf water relations and hydraulics, especially under high light, suggesting that warming constrains the range of physiological responses available to cope with drought (Figure 5). Under shade, warming enhanced the plasticity of some traits, particularly those related to gas exchange. This suggests a strategy shift under low light, where T32 plants adopt a more acquisitive phenotype (e.g., a higher number of cheaper leaves with lower *LMA*) to offset reduced carbon gain. However, this came at the expense of hydraulic safety, as shown by reductions in *K*_L_, *H*_v_, and *SM*_leaf_, emphasizing the trade-offs induced by warming. The overall *PPi* for all treatments and variables is presented in Figure A6.

**Figure 6:**
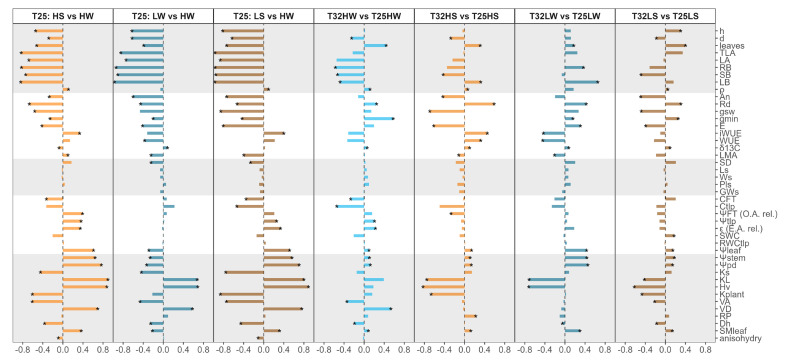
Plasticity score of each trait to each growing factor. From left to right, the first three columns analyze the effect of water deficit at high light (HS vs. HW), the effect of light intensity under well-watered conditions (LW vs. HW), and the combined effect of water deficit and shade (LS vs. HW) at T25. The next columns reflect the effect of growing temperature under well-watered conditions and high light intensity (T32HW vs. T25HW), the effect of growing temperature under water deficit conditions and high light intensity (T32HS vs. T25HS), the effect of growing temperature under well-watered conditions and shade (T32LW vs. T25LW), and finally, the effect of growing temperature under water deficit and shade (T32LS vs. T25LS). A negative plasticity score (NP, ranging from −1 to 0, see Materials and Methods for equation) was used when the first treatment (the one on the left side of the comparison) had lower values than the reference treatment (the one on the right side) and is referred to mean values of the reference treatment. By contrast, the positive plasticity score (PP ranging from 0 to 1) uses the difference between the treatment and the reference mean but is divided by the treatment mean. Asterisks indicate significant differences at *p* < 0.05 by *t*-test between each growing condition. Note that water potentials were considered positive here, hence positive plasticity (PP) means lower water potential and also lower SMleaf.

The degree of plasticity differed among trait categories. Growth-related traits and biomass partitioning were among the most plastic across environments. (Figure 6). In contrast, wood density (ρ) showed little plasticity, although it was significant for many treatments, including an increase due to water deficit or warming (Figure 6). Stomatal morphology showed limited plasticity, and S_D_ was more plastic than L_s_. Among the most temperature-sensitive traits were *g*_min_, Cπ_0_, *H*_v_, *V*_D_, *K*_L_, *k*_plant_, number of leaves, and root biomass (Figure 6).

### 2.6. Correlations Among Traits and Principal Components Analysis (PCA)

**Figure 7:**
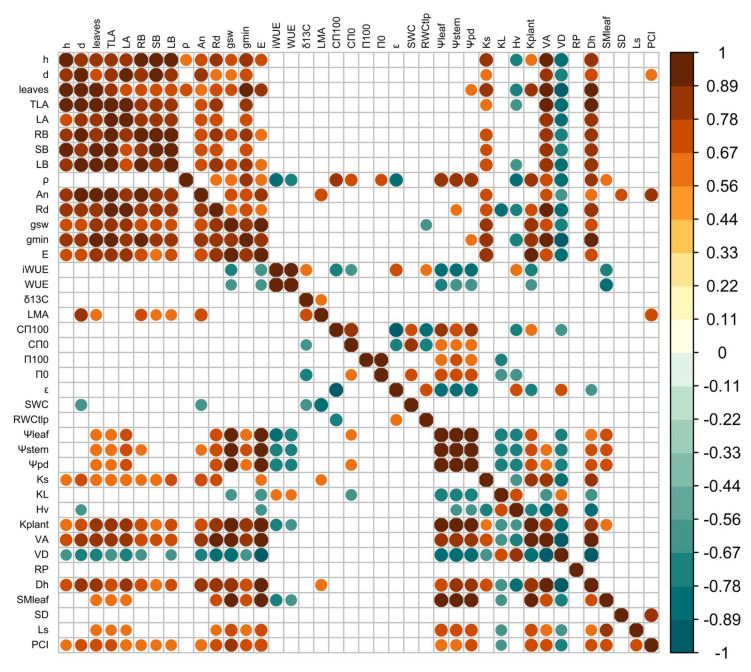
Pearson’s correlation matrix for traits in plants growing at T25 (upper triangle) and at T32 (lower triangle). Significant correlations are color-coded, with positive correlations in warm colors and negative correlations in cool colors. See Table A1 for abbreviations.

Warming substantially altered the coordination among functional traits in *Fagus sylvatica* seedlings. Some traits showed strong positive correlations at both temperatures: plant growth traits (height and diameter) with *A*_n_, *K*_s,_ and *V*_A_ (Figure 7). At T25 we found significant positive correlations between plant height and ρ and trade-offs between height and *H*_v_, *ρ* and *H*_V,_ and *ρ* and *iWUE*. In contrast, at T32, many of these correlations weakened or changed direction, suggesting a restructuring of trait integration in response to warming (Figure 7). For example, at T32, plant diameter was significantly correlated with *LMA* and k_plant_. At T25, high *iWUE* negatively correlated with *g*_sw_, *ρ*, leaf *C*п_100_, *k*_plant_, leaf water potentials, and *V*_D_, and positively correlated with *δ^13^C*, *ε*_leaf_, *RWC*_п0,_ and *H*_v_. However, at T32, *iWUE* became decoupled from most hydraulic traits and only retained weak negative correlations with *g*_sw_ and leaf water potentials. This indicates that warming disrupted the typical trade-offs and synergies that underlie water-use strategies. Similarly, stomatal length (L_s_) showed significant correlations with multiple traits at T32, including number of leaves, *g*_sw_, *g*_min_, and *SM*_leaf_, relationships that were absent or weaker at T25 (Figure 7). This suggests that under warming, stomatal traits may gain importance as integrative regulators of leaf-level water dynamics and thermal regulation.

**Figure 8:**
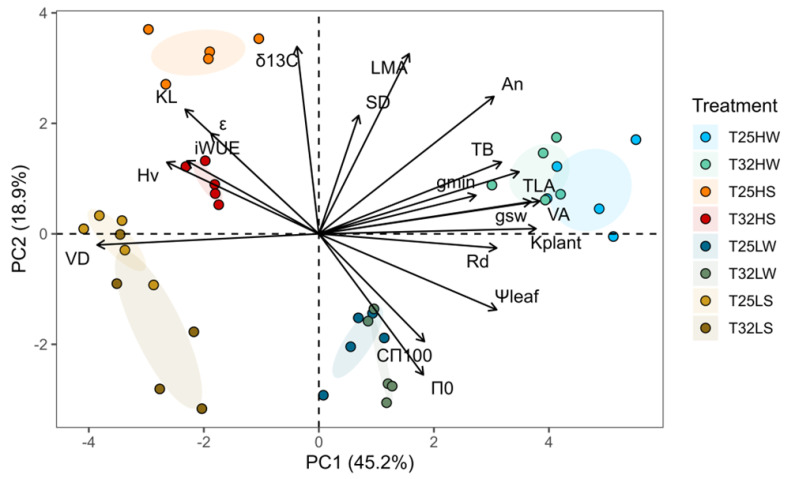
Principal Component Analysis (PCA) for the evaluated traits. Arrows represent the eigenvectors of each variable for the first two principal components (PC1 and PC2). The direction and length of the arrows indicate the contribution and importance of each variable to the principal components. Each plant is represented by a different color depending on the treatment. The oval areas represent the centroids of each treatment ± 75% of the variation within the treatment.

Principal Component Analysis (PCA) further clarified how temperature modulated the main axes of phenotypic variation (Figure 8). The first component (PC1, 45% of variance) was associated with water availability, separating well-watered and water-stressed plants. Traits with high positive loadings on PC1 included *k*_plant_, *V*_A_, *g*_sw_, *g*_min_, *Ψ*_leaf_, and *TLA*, reflecting efficient water transport and growth. Negative scores were associated with traits favored under drought, such as *iWUE*, *V*_D_, *K*_L_, *H*_v,_ and *ε*_max_. The second component (PC2, 19% of variance) reflected light intensity effects. Traits with positive loadings include *LMA*, *δ^13^C*, *A*_n_, and *S*_D,_ while negative loadings include *π*_0_ and *C*_π100_. Importantly, warming shifted the position of treatments in PCA space, particularly under water stress. For example, T32 HS plants were further displaced from the HW quadrant, driven by reduced *k*_plant_, *Ψ*_leaf_, and *A*_n_ (Figure 8).

## 3. Discussion

Temperature is one of the primary drivers of plant function and development. However, compared to other environmental stressors such as drought or shade, the direct effects of elevated temperature on tree physiology and performance remain relatively understudied. The present study is among the first to isolate the direct effect of growth temperature on key morphological, physiological, and anatomical traits in forest tree seedlings while controlling for vapor pressure deficit (VPD). Furthermore, we address a gap of knowledge by examining how warming interacts with concurrent stressors such as water deficit and shade in tree species, within the framework of “multifactorial stress combination”, a concept recently explored mainly in model plant species [56]. Our findings show that warming exerted a direct and predominantly negative effect on several functional traits related to plant growth and physiology (Figure 6), reduced phenotypic plasticity, especially in response to drought, and impaired certain acclimation responses observed at temperatures close to the optimum (T25) that were absent at supraoptimal temperatures (T32). Nevertheless, *F. sylvatica* seedlings showed some adaptive strategies that may facilitate acclimation to warming. In the following sections, we discussed the extent of plant acclimation to warming and how warming may have affected the shade and drought tolerance of this late-successional species. 

### 3.1. Impact of Warming on Plant Morphology, Physiology, and Anatomy at High and Low Irradiance

Warming consistently reduced plant biomass and stem diameter (Figure 1), whereas height was less affected or even enhanced under shade, consistent with previous studies of trees growing near their warmer range limit [57,58]. Wood density (ρ) increased at T32, likely reflecting an investment in mechanical support and xylem safety [59]. Anatomically, warming increased *V*_D_ and decreased *V*_A_ at high irradiance (Figure 4), supporting the formation of denser wood with narrower conduits. These anatomical changes can hamper hydraulic conductivity and efficiency of water transport but limit the spread of xylem embolism. These anatomical changes suggest a trade-off between hydraulic efficiency and safety, consistent with previous studies in warming-exposed hardwoods. [60,61]. Importantly, *K_s_* was not significantly altered by warming alone (Figure 4), indicating that the physical properties of sap (e.g., reduced viscosity) may partially buffer xylem conductivity under higher temperatures [62], as observed in trembling aspen [29]. Our results highlight the acclimation capacity of beech seedling xylem to warming, thereby supporting our third hypothesis. This contrasts with the limited xylem adjustment of gymnosperms exposed to prolonged experimental warming [28]. 

At high irradiance, warming (T32 HW) decreased total leaf area (*TLA*), leaf mass per area (*LMA*), and leaf size (*LA*), despite an increased number of leaves (Figure 1). These morphological adjustments likely facilitate thermal regulation by reducing the boundary layer thickness and promoting evaporative cooling [40,63]. The same pattern was observed in warm-adapted *Populus* trees, which down-regulated leaf temperature by producing smaller and thinner leaves with higher stomatal density [64]. Hence, warming promoted a conservative production of transpiring tissue under high irradiance, which correlated with a concomitant decrease in *V*_A_, indicating a close correlation between plant transpiration rate and water supply [65]. The reduction in the net assimilation rate (*A*_n_) by 15% under warming, coupled with a 25% increase in respiration (*R*_d_), confirms the cost of elevated temperature on carbon balance and supports our first hypothesis (Figure 2 and Figure A2). The temperature optimum for *A*_n_ in beech was estimated at 24.5 °C [66]; hence. the slight reduction of *A*_n_ at 32 °C could be attributed to thermal acclimation, possibly by raising the *A*_n_ optimum temperature at T32 [9,19]. In any case, the depletion of *A*_n_ might be associated with metabolic constraints, particularly the downregulation of leaf photochemistry, which frequently occurs under elevated temperatures [67], rather than diffusional limitations to CO_2_. Increased stomatal conductance (*g*_sw_) and transpiration (*E*) at T32, even under constant VPD, demonstrate a shift toward evaporative cooling mechanisms to mitigate leaf overheating [54]. This rise in *g*_sw_ and *E* correlated also with higher *k*_plant_, suggesting strong coordination between water supply and demand at the plant level. Higher temperatures also promoted faster water use and higher leaf minimum conductance (*g*_min_), which eventually led to faster depletion of soil water for warmed plants [68]. Thus, reduction in *TLA* at T32 HW was important to avoid lowering *Ψ*_leaf_, beyond the leaf safety margin (*SM*_leaf_) since *π*_0_ did not change with temperature. Additionally, the reduced *LMA* observed in warmed plants limited their leaf capacitance before or after turgor loss (Figure 6), partially aligning with previous findings [29].

Growing under shaded conditions, when water was not limited (LW), mitigated the negative effects of warming described above and stimulated plant growth and biomass. Indeed, T32 LW seedlings produced greater biomass, higher wood density, more leaves, and showed increased allocation to leaf tissue with lower *LMA* (Figure 1 and Figure A3), suggesting an acquisitive strategy adapted to low light and thermal stress, aimed to compensate for the reduced *A*_n_ but higher *R*_d_. These plants prioritized maximizing *TLA* and transpiration, including g_min_, to maintain CO_2_ diffusion, increase *C*_i,_ and reduce leaf temperature (Figure 2 and Figure 4), in a way to minimize photorespiration losses [69], at the cost of reduced hydraulic safety. Indeed, *K*_L_, *H*_V,_ and *SM*_leaf_ were significantly lower, indicating increased vulnerability to water stress. Regarding changes in *g*_min_, at moderately high temperatures (<40 °C), the stomatal component of *g*_min_ is of greater importance than at higher temperatures and VPD, where the cuticle component governs g_min_ [70]. Accordingly, we found strong correlations between *g*_min_ and stomatal traits, especially *PCI*, which suggested a lack of complete closure of the stomata as the main cause explaining the rise in *g*_min_ at T32. On the other hand, T25 LW followed a more conservative strategy, with lower *TLA* and gas exchange, but higher *Ψ*_leaf_ and tougher leaves (high *LDMC* and *LMA*). This fast-growing strategy of T32 LW could be risky if water is limited, as it features a higher leaf area but reduced leaf capacitance and *Ψ*_leaf_, which lowers *SM*_leaf_ (Figure 3). This makes beech-shaded phenotypes more vulnerable to water stress under warming [71]. 

### 3.2. Impact of Warming and Water Deficit at High and Low Light Intensity

The interaction between elevated temperature and water deficit imposed significant constraints on the hydraulic functioning and carbon economy of *Fagus sylvatica* seedlings. Seedlings grown under T32 with limited water availability (HS and LS treatments) showed pronounced reductions in *A*_n_, *LA*, *TLA*, *LMA,* and *g_sw_*, while simultaneously exhibiting increased respiration and reduced osmotic adjustment capacity (Figure 6). 

As water availability diminishes, elevated growth temperatures lead to a steeper reduction in *A*_n_, primarily influenced by increased stomatal closure to avoid hydraulic dysfunction but at the cost of decreased carbon uptake, increased hydraulic resistance, and growth [38] (Figure 2 and Figure 4). Reduced *A*_n_ in water-stress T32 plants may also arise from lowered synthesis of protective enzymes, antioxidants, and heat dissipation proteins, essential for mitigating the higher thermal and oxidative stress produced by limited leaf cooling capacity [72]. Furthermore, water-stressed plants showed lower *K*_s_ but a more redundant xylem, consistent with a strategy aimed at damage mitigation rather than efficiency [61]. The start of embolism formation in *F. sylvatica* was estimated at *Ψ*_stem_ below -1.8 MPa [73,74], although beech seedlings from the same population and acclimated to water deficit reached this point at much lower levels (−2.4 MPa), suggesting a very narrow xylem safety margin to preserve xylem functioning under drought [74]. Lower *Ψ*_stem_ and *Ψ_leaf_* in T32 water-stressed plants may be the consequence not only of higher biomass allocation to leaves and decreased water viscosity [75] but also of a lack of leaf osmotic and elastic adjustments. The limited osmotic adjustment, together with the lower *Ψ_leaf_,* decreased *SM_leaf_* to almost 0.2 MPa. A lack of thermal acclimation was also observed for leaf capacitance and *ε_max_*, decreasing further the plant’s ability to tolerate fluctuations in *Ψ_leaf_* and maintain turgor pressure (Figure 3) [74]. Interestingly, shade intensified the negative effects of combined warming and water stress. T32 LS plants exhibited the lowest values of *K*_L_, *H*_V_, *k_plant_*, and *SM_leaf_*, indicating an impaired ability to supply water to leaves and protect against desiccation. Although shaded seedlings generally displayed an acquisitive strategy under warming when water was available, this response became maladaptive under concurrent drought, leading to decreased performance and potential mortality risk [76].

In contrast, T25 plants under drought showed more effective acclimation strategies. They were able to double *A*_n_ of T32 HS, achieve more than 0.8 MPa of active osmotic adjustment, lowering *Ψ*_п0_ below 3 MPa (Figure 2 and Figure 3; Table A3), and increasing *ε_max_.* T25 HS also showed higher leaf shedding, which increased *H*_V_ and the supply of water to the remaining leaves (higher *K*_L_), lowering vessel area and producing more redundant xylem (Figure 3; [77]), overall improving the whole-plant water balance [78] and sustaining higher leaf gas exchange rates (Figure 2) [23,79]. Both temperature treatments decreased *g*_min_ in response to water deficit, which may offer significant advantages for the conservation of xylem function, particularly during warmer and drier conditions [23,79].

Collectively, these findings reveal that warming exacerbates the negative effects of drought and that while moderate drought or warming alone can be tolerated through structural or physiological adjustment, their combination imposes compounded stress that exceeds the plastic limits of key traits. The results underscore the importance of trait coordination in stress resilience and suggest that *F. sylvatica* seedlings may face severe regeneration challenges under future climate scenarios characterized by higher temperatures, reduced soil moisture, and light limitation beneath forest canopies.

### 3.3. Phenotypic Plasticity and Regeneration Niche of Beech Seedlings Under Multi-Stress Factors

**Figure A1:**
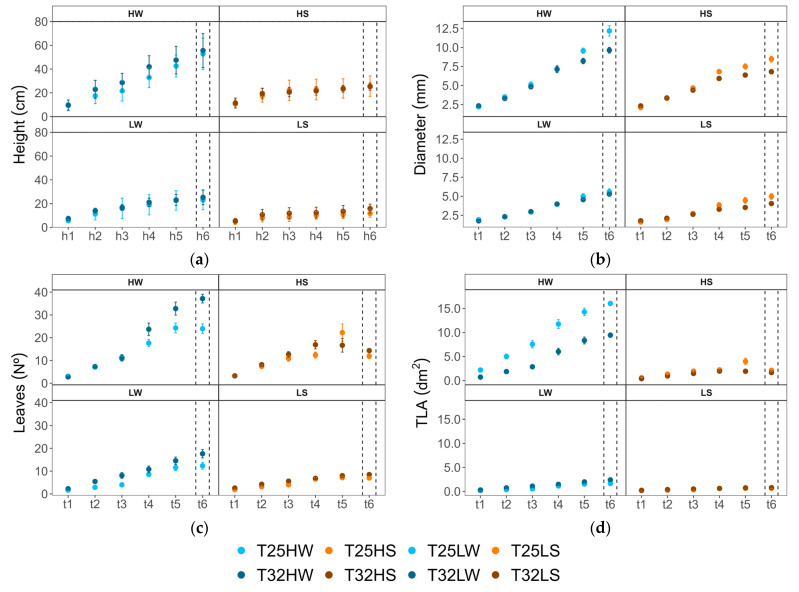
Temporal dynamics of plant morphological traits: (**a**) height (h, cm), (**b**) diameter (d, mm), (**c**) leaves number (Leaves, no.), and (**d**) Total leaf area (*TLA*, cm^2^) measured over six different assessments (h1–h6, d1–d6, L1–L6, and LA1–LA6, respectively) to different treatments: HW (high light well-watered), HS (high light water-stressed), LW (low light well-watered), LS (low light water-stressed) and growth temperatures (25 °C, T25, and 32 °C, T32). Data points represent means ± standard error (SE). Dashed vertical lines indicate the final measurement point, corresponding to the moment of sampling and measurement for the results discussed in this study.

**Figure A2:**
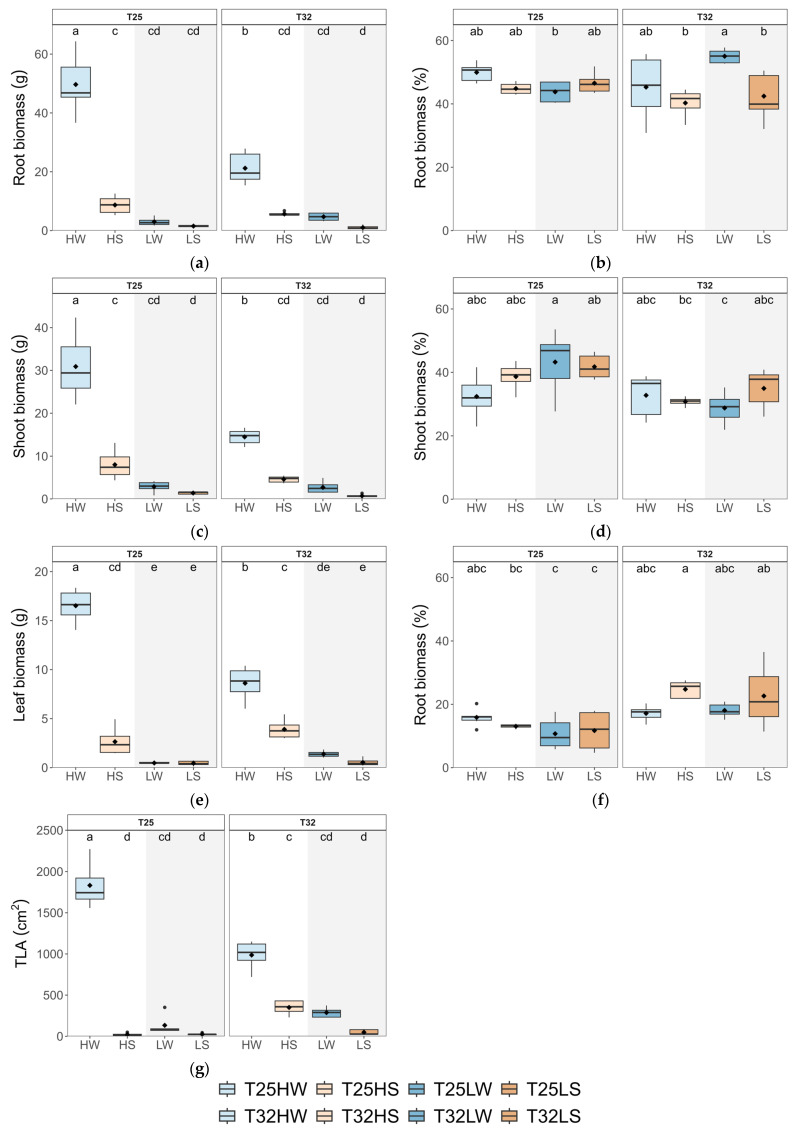
Boxplots of biomass traits and of beech seedlings grown in a factorial combination of temperature: 25 °C (T25) vs. 32 °C (T32), soil water availability: well-watered (W) vs. water-stressed (S), and light intensity: high (H) vs. low (L): (**a**) absolute root biomass (RB, g); (**b**) relative root biomass to total biomass (RB, %); (**c**) absolute shoot biomass (SB, g); (**d**) relative shoot biomass to total biomass (SB, %); (**e**) absolute leaves biomass (LB, g); (**f**) relative leaves biomass to total biomass (LB, %) and (**g**) total leaf area (TLA, cm^2^). The straight horizontal black line within each boxplot indicates the median, while the mean value is represented as a solid black point. Different letters indicate significant differences among treatments.

**Figure A3:**
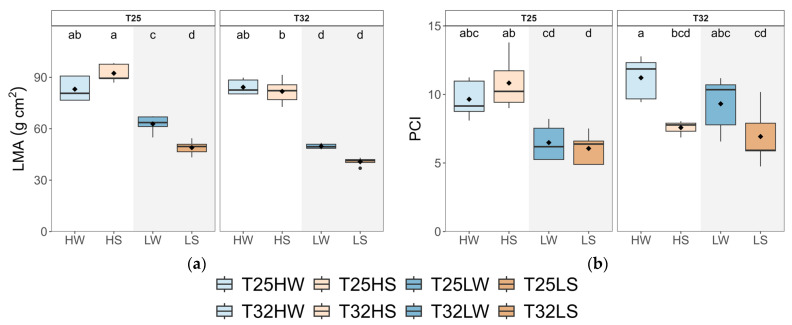
Boxplots of leaf morphological traits of beech seedlings grown in a factorial combination of temperature: 25 °C (T25) vs. 32 °C (T32), soil water availability: well-watered (W) vs. water-stressed (S), and light intensity: high (H) vs. low (L): (**a**) leaf mass area (LMA, kg m^−2^); (**b**) potential conductance index (PCI). The straight horizontal black line within each boxplot indicates the median, while the mean value is represented as a solid black point. Different letters indicate significant differences among treatments.

**Figure A6:**
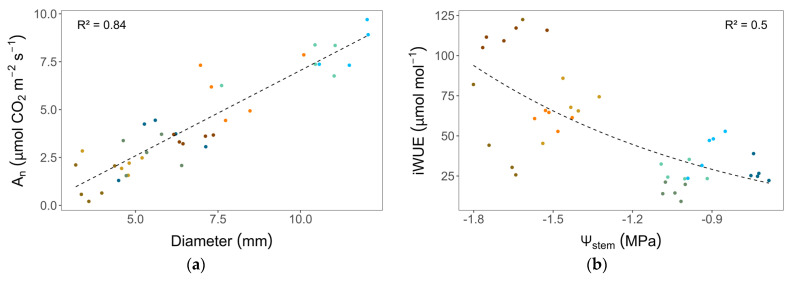

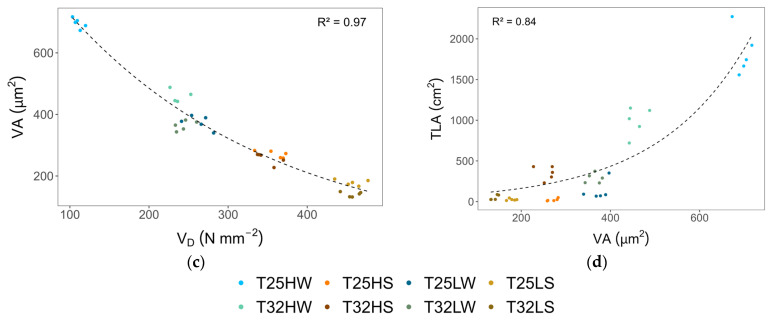
Relationships between functional traits: (**a**) strait line regression between net photosynthesis (A_n_) and stem diameter (**d**); (**b**) Quadratic (second-order polynomial) regression between intrinsic water use efficiency (iWUE) and stem water potential at midday (Ψ_stem_); (**c**) Negative quadratic relationship between vessel density (V_D_) and mean vessel cross-sectional area (V_A_) and (**d**) Positive quadratic relationship between vessel area (V_A_) and total leaf area (TLA). Colored symbols represent different treatments: HW (high light well-watered), HS (high light water-stressed), LW (low light well-watered), LS (low light water-stressed) and growth temperatures (25 °C, T25, and 32 °C, T32). Dashed lines indicate the fitted regressions; coefficients of determination (R^2^) are reported in the upper corner of each panel.

**Figure A7:**
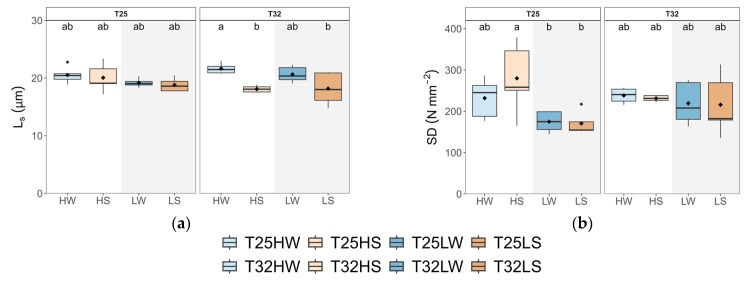
Boxplots of stomatal traits of beech seedlings grown in a factorial combination of temperature: 25 °C (T25) vs. 32 °C (T32), soil water availability: well-watered (W) vs. water-stressed (S), and light intensity: high (H) vs. low (L): (**a**) stomatal length (L_s_, μm) and (**b**) stomatal density (SD, number of stomatas per mm^−2^). The straight horizontal black line within each boxplot indicates the median, while the mean value is represented as a solid black point. Different letters indicate significant differences among treatments.

A central finding of this study is that elevated temperature reduced the phenotypic plasticity of key functional traits in *F. sylvatica*, particularly under stressful combinations of drought and shade. These results are in agreement with our fourth hypothesis related to the cost associated with maintaining plasticity in resource-limited environments [80]. The calculated plasticity index (*PPi*) showed clear reductions at T32 for traits involved in hydraulics, leaf water relations, and growth, indicating a narrowing of the seedlings’ capacity to respond to environmental heterogeneity (Figure 5 and Figure 6). These reductions were especially pronounced under drought, suggesting that warming compromises acclimation to water limitation. Phenotypic plasticity allows plants to modify their phenotype to match their current environment. However, there are limits and costs associated with plasticity [81]. We show that plasticity was trait- and context-dependent probably due to the complex interaction among traits. Traits related to whole-plant biomass showed greater plasticity than those measured at the organ or tissue levels (Figure 5). While leaf gas exchange retained moderate plasticity under warming, hydraulic and anatomical traits were less flexible. Wood density (*ρ*), xylem anatomy (*V_A_*, *V_D_*), and stomatal morphology exhibited minimal plastic responses, implying structural constraints (Figure 6 and Figure A1) and indicating higher costs [82]. Interestingly, warming in the absence of water deficit enhanced plasticity at low light intensity (Figure 6), enabling a compensatory increase in leaf number and area and revealing the strong adaptation of this shade-tolerant species to grow in the humid forest understory [83,84]. However, this strategy was not viable under drought, revealing a trade-off between plasticity for carbon acquisition and vulnerability to hydraulic failure. Extensive phenotypic plasticity in key functional traits is often considered favorable for the persistence of populations under rapid climate change [3]. Nevertheless, our results emphasize that plasticity itself is temperature-sensitive and that warming may push plants beyond the threshold where plastic responses remain beneficial. The reorganization of trait correlations under warming (as revealed by PCA and correlation analysis) further suggests that trait integration shifts under stress, potentially limiting coordinated responses. 

From an ecological perspective, these findings raise concerns about the capacity of *F. sylvatica* to regenerate in future forests, particularly near its southern distribution limit [37,85,86]. Reduced plasticity under warming may hinder seedling establishment in the face of increasing drought and shade. Moreover, if warming continues to suppress osmotic adjustment, reduce safety margins, and destabilize hydraulic architecture, even short drought periods could compromise regeneration success. Understanding these physiological thresholds is critical to forecasting forest dynamics under climate change and informing management strategies for sensitive late-successional species like European beech.

## 4. Materials and Methods

### 4.1. Plant Material, Growth Conditions, and Treatments

Beech seedlings were established from nuts collected in the Natural Reserve of ‘Hayedo de Montejo’ in 2021 (458,249; 4,551,615 UTM ETRS89). In February 2022, after three months at 4 °C in darkness and moist conditions, one seed per pot germinated within a few days of difference in 9 L pots, filled with a mixture of peat and sand (75–25% v:v). Each pot was fertilized with a release fertilizer (Nutricote^®^, 16N:4.4P:8.3K, 2.5 gr L^−1^ of substrate). Pots were randomly arranged in two different walk-in growth chambers set at two different temperatures but with the same air *VPD*. One chamber was set with day/night temperatures and relative humidity of 25/15 ± 0.5 °C and relative humidity of 40/68 ± 1% (VPD of 1.91/0.86 ± 0.06 kPa) (named T25), close to the temperature optimum for photosynthesis for beech [66], and the other to 32.5/22.5 ± 0.5 °C and 61/80 ± 1% (VPD of 1.91/0.86 ± 0.06 kPa) (named T32), both with a 12 h of photoperiod. A +7.5 °C increase in mean temperature is expected from June to August for Western and Central Europe and the Mediterranean region according to the scenario SSP5-8.5 for 34 models of AR6 CMIP6 (interactive-atlas.ipcc.ch) [1]. In each chamber, we established two treatments of light intensity: high PPFD (ca. 600 μmol photons m^−2^ s^−1^ at the canopy height) and low PPFD (ca. 200 μmol photons m^−2^ s^−1^ at the canopy height). Low PPFD (e.g., shaded conditions) was provided by gray polyethylene shade nets mounted on frames and was measured as the average of six different measurements using a quantum meter (MQ 200; Apogee Instruments Inc., Logan, UT, USA) in a horizontal position on leaves. Plants developed under well-watered conditions for two months when seedlings with similar height and number of leaves were assigned to two contrasted watering treatments. Well-watered plants were watered daily to full capacity. Water-stressed plants were kept without watering until stomatal conductance reached 1/3 of well-watered plants (ranging from ca. 10 days in high PPFD to ca. 24 days in low PPFD) and then plants were watered every three days with an initial amount of 150 mL of water for plants under high PPFD and 65 mL of water for plants under low PPFD (about 1/3 of daily evapotranspiration in both cases). Once a week, plants were weighed and the volumetric soil water content (*SWC*) was measured by time domain reflectometry (TDR, Trase System I, Soil Moisture Equipment, Logan, UT, USA) and watering amounts were adjusted. At this time the final multifactorial design (two factors: water and light) was set for each growing temperature: (1) high PPFD (ca. 600 μmol photons m^−2^ s^−1^ at the canopy height) and well-watered conditions (HW); (2) high PPFD and moderate water stress (HS); (3) low PPFD (ca. 200 μmol photons m^−2^ s^−1^ at the canopy height) and well-watered conditions (LW); (4) low PPFD and moderate water stress (LS). Each week, plants were randomly repositioned within their treatment group. Every three weeks, plants were rotated between chambers while maintaining their assigned warming treatment (i.e., identical conditions but a different growth chamber)

### 4.2. Growth and Biomass Allocation

Growth and biomass were measured in six randomly selected plants of each treatment at the beginning of the experiment. These plants were not used for any other measurement until harvest. Height, stem diameter, and the number of fully developed leaves were measured six times throughout the experiment (every 15–20 days) (see Figure A1). After 90 days under their assigned treatments, the same plants were harvested, and biomass was divided into roots, stems, and leaves. Roots were separated from the soil by gently washing under clean tap water. Samples were oven-dried at 60 °C for 7 days and weighed. We calculated root (*RM*), stem (*SM*), and leaf mass (*LM*) fractions as the ratio of organ dry mass to total plant dry mass. One representative fully mature leaf from the top canopy of each plant was scanned to obtain the leaf area using ImageJ 1.53t (NIH, Bethesda, MD, USA) [87] and then oven-dried at 70 °C for 48 h for constant dry mass (Mettler Toledo AB 204, Columbus, OH, USA) to determine the leaf mass per area (*LMA*) as the ratio of leaf dry mass to projected area. The total leaf area (*TLA*) of each plant was estimated from *LM* and *LMA*. The mean leaf area for each plant (*LA*) was calculated as *TLA* divided by the number of leaves.

### 4.3. Gas Exchange and Water Use Efficiency

One fully mature leaf from the upper plant canopy was selected to measure the net photosynthetic rate (*A*_n_, μmol CO_2_ m^−2^ s^−1^), stomatal conductance (*g*_sw_, mol H_2_O m^−2^ s^−1^), and transpiration rate (*E*, mol H_2_O m^−2^ s^−1^). These parameters were measured twice during the experiment in five plants per treatment using a portable IRGA system (LI-6400XT) with the transparent chamber (6400-08 clear chamber) to record the leaf gas exchange under growth conditions. Measurements were conducted under growth conditions of air temperature (block temperature was set at 25 °C for T25, and 32 °C for T32) and relative humidity to achieve a VPD close to 1.9 kPa. The reference CO_2_ concentration was set at 400 μmol m^−2^ s^−1^ and a flow rate of 300 μmol m^−2^ s^−1^. After recording gas exchange under growing conditions, the light source was switched off inside the chambers, and after 20 min of acclimation in the dark, dark respiration (*R*_d_) was measured. All gas exchange variables were corrected with the minimum conductance (*g*_min_) (see below) according to [88]. The leaf water use efficiency (*WUE*) was calculated as (*A*_n_/*E*) and the intrinsic water use efficiency (*iWUE*) was determined using (*A*_n_/*g*_sw_).

### 4.4. Stomatal Traits and Carbon Isotope Composition (δ^13^C)

The same leaves used for gas exchange measurements were collected for carbon isotope composition (*δ^13^C*) and then for stomatal traits. Two leaf disks taken from each side of the leaf blade were dried for 3 days at 65 °C and analyzed at the Stable Isotope Facility of UC Davis (CA, USA). Isotope composition was measured with an elemental analyzer (PDZ Europa ANCA-GSL, Sercon Ltd., Cheshire, UK) interfaced to an isotope ratio mass spectrometer (PDZ Europa 20-20; Sercon Ltd., Cheshire, UK) with internal standards reaching δ^13^C_VPDB_ standard deviation <0.07‰. Leaf trichomes were removed using duct tape and three negative impressions were taken of the middle portion of the leaf avoiding main veins and using nail varnish. The impressions were attached to a microscope slide using transparent tape and imaged under a light microscope (Leica DM2500 LED) using the software Leica Application Suite X 3.7.2.22383 (Leica Microsystems GmbH, Wetzlar, Germany). Photomicrographs were analyzed using ImageJ [87]. Stomatal density (*S*_D_) was calculated as the number of stomata per unit leaf area (nº mm^−2^) on three leaf areas of 1.15 mm^2^ each using ×10 magnification. Within each leaf area, 10 stomata were randomly selected to measure in μm the guard cell length (*S*_L_), stomatal pore length (*Pl*_s_), stomatal complex width (*W*_s_) and guard cell width (*GW*_s_) using ×40 magnification. The potential conductance index (*PCI*) was computed as PCI = (*GW*_s_)^2^ SD 10^−4^ [89], as a surrogate of potential maximum stomatal conductance.

### 4.5. Minimum Leaf Conductance or Residual Conductance (g_min_)

At the same time as gas exchange measurements, one healthy, fully expanded leaf from the same plants was immediately stored in a plastic zip bag with moist tissue paper and kept in a portable cooler at 4 °C until it was taken to the laboratory. In the lab, leaves were stored at 4 °C in darkness with the petiole submerged in water overnight until the next day for measurements. We estimated *g*_min_ through bench dehydration of the rehydrated leaves with petioles sealed with a high-strength adhesive (Loctite, Prism 401) [90]. Briefly, the leaf area was measured at the beginning of dehydration, and leaves were placed on top of a perforated rack at constant air temperature and relative humidity (ca. 25 °C and 46%, respectively) without direct light. They were repeatedly weighed with an analytical balance to determine the steady-state rate of water lost by unit of leaf area once stomata were closed, i.e., epidermal transpiration (*E*_min_, $mol\;H_{2}O\;m^{-2}\;s^{-1}$). To achieve the epidermal minimum conductance by one side of the leaf (*g*_min_, $mol\;H_{2}O\;m^{-2}\;s^{-1}$), we followed the next approximation (*g*_min_ = *E*_min_ P/(2 VPD)), where P is the atmospheric pressure (94 kPa) and VPD is the air vapor pressure deficit (1.65 kPa), and the boundary layer conductance was assumed negligible.

### 4.6. Pressure-Volume Curves and Related Traits

Leaf water relations were measured using pressure-volume (P-V) curves of leaves sampled at the end of the night period on five seedlings per treatment. Leaves were rehydrated for six hours in the dark and at 4 °C with only the petiole immersed in distilled water. After that, leaves were scanned to obtain the initial leaf area and were allowed to slowly dehydrate at constant room temperature using the free transpiration method [91,92]. Leaf water potential (*Ψ*_leaf_) was measured with a pressure chamber (model 1000; PMS Instrument Co., Albany, NY, USA) and leaf mass with a precision balance at intervals, starting with Ψ_leaf_ > −0.05 MPa and until ca. −3.5 MPa for HW and LW treatments and −6 MPa for HS and LS treatments. We checked all curves for oversaturation during the first steps of dehydration and when present was corrected [93,94]. Leaf dry mass was determined, as described above. The osmotic potential at full turgor (*π*_100_), the turgor loss point (*π*_0_), leaf capacitance before the turgor loss point (*C*π_100_) and after (*C*π_0_), the maximum modulus of elasticity (ε_leaf_), and the relative water content at the turgor loss point (*RWC* π_0_) were derived from the P-V curves according to [91,95].

### 4.7. Predawn and Midday Water Potentials

The same leaves used for gas exchange measurements were used to determine midday leaf water potentials (*Ψ*_leaf_) after cutting the petiole and sealed inside a plastic bag that had been exhaled and stored in ice boxes. They were then transported to the lab and measured with a pressure chamber (PMS instrument; model 1505D) within 1–2 h of excision [96]. One adjacent leaf was previously covered and allowed to equilibrate for at least 3 h before midday with cling wrap and aluminum foil to prevent transpiration, and to determine the midday branch water potential (*Ψ*_stem_) (i.e., non-transpiring leaf). Leaf predawn water potential (i.e., end of the night period) (*Ψ*_pd_) was measured the night before on the same plants. The safety margin (*SM*_leaf_) was calculated as the absolute difference between the turgor loss point (π_0_) and the transpiring leaf water potential at midday (*Ψ*_leaf_). 

### 4.8. Hydraulic Conductivity and Huber Value

Loss of hydraulic conductivity and maximum hydraulic conductivity were measured in nine seedlings per treatment at the end of the experiment, following [97]. Plant transpiration was stopped 3 h before harvesting by switching off the lights and enclosing plants in black bags. Then, pots were immersed in water and the stem was cut at the base. The entire above-ground portion was kept under water, tearing leaves away from shoots, and the plant distal end was cut to ensure xylem relaxation for 30 min [74]. The stem was then sequentially cut back underwater, and a 5 cm long segment above the cotyledons was connected to a XYL’EM apparatus (Bronkhorst, Montigny les Cormeilles, France) to measure hydraulic conductivity at low pressure (≤2 kPa) before (*K*_init_) and after flushing the sample with degassed, filtered 2 mmol KCl solution at high pressure (0.2–0.3 MPa) for 20 min (*K*_max_). Loss of hydraulic conductivity (*PLC*) was determined as:

(1) $$PLC=100(1-K_{init}/K_{max})$$

Maximum specific hydraulic conductivity (*K*_s_) was calculated by dividing *K*_max_ by the sapwood area and leaf-specific conductivity (*K*_L_) by dividing *K*_max_ by the total supported leaf area. The Huber value (*H*_V_) was determined as the ratio of *K*_L_ and *K*_s_ (i.e., sapwood area relative to leaf area). Finally, the hydraulic conductance of the whole plant (*k*_plant_) was estimated as in [98]:

(2) $$k_{plant}=\frac{E}{\left(\Psi_{pd}-\Psi_{leaf}\right)}$$

where *E* was the leaf transpiration rate and (*Ψ*_pd_ − *Ψ*_leaf_) was assumed to be the water potential gradient from the soil to leaf (MPa), assuming full equilibration of plant and the soil at the end of the night period (i.e., *Ψ*_soil_ ≈ *Ψ*_pd_).

### 4.9. Stem Xylem Anatomy

Five stem segments per treatment, those used for hydraulic conductivity measurements, were selected for anatomical measurements. One centimeter in length was used to determine the wood density (ρ) following [74]. Fifteen µm-thick cross and tangential sections were cut using a sliding microtome (Leica SM 2400), stained with 0.1% safranin, and mounted for subsequent image analysis. Digital images of the thin sections were taken using a Moticam A1 camera (Motic, Hong Kong, China) attached to an Olympus BX50 light microscope. Images were analyzed using ImageJ [87]. We measured three sectors per sample from the cross-section to quantify the average vessel lumen area (*V*_A_), vessel density (*V*_D_), the equivalent circle diameter (*D*), and the hydraulic diameter (*D*_h_) following [99]:

(3) $$D=\sqrt{\frac{4V_{A}}{\pi }}$$

(4) $$D_{h}={\left(\frac{\sum D^{4}}{N}\right)}^{\frac{1}{4}}$$

where *V*_A_ is the lumen conduit area, *N* it is the number of vessel elements. To determine the parenchyma surface area, a further three sectors per sample were taken from the tangential section.

### 4.10. Trait Variation and Plasticity

We calculated the effect of each treatment individually by expressing the variation in each measured trait (see Table A1 for definitions) as a normalized value ranging from −1 to 1. Negative values indicate a decrease in the trait as a consequence of the treatment, while positive values indicate an increase. When a trait changes—either increasing or decreasing—compared to a reference value, the following formulas were used to quantify plasticity:

If the treatment mean of a variable (μ_treatment_) < reference mean (μ_reference_), the plasticity is negative:

(5) $$NP=\frac{\left(\mu_{treatment}-\mu_{reference}\right)}{\mu_{reference}}$$

If the mean of treatment (μ_treatment_) > the mean of reference (μ_reference_), the plasticity is positive:

(6) $$NP=\frac{\left(\mu_{treatment}-\mu_{reference}\right)}{\mu_{treatment}}$$

Note that we can obtain the individual trait response ratio as:

(7) $$\frac{\mu_{treatment}}{\mu_{reference}}=NP+1$$

Or:

(8) $$\frac{\mu_{treatment}}{\mu_{reference}}=\frac{1}{1-PP}$$

where *NP* and *PP* are the values of the negative or positive plasticity for the trait, respectively.

The Phenotypic Plasticity Index (*PPi*) was calculated to assess the overall plasticity of *Fagus sylvatica* seedlings to different environmental conditions, evaluating the effects of growth temperature on light and water plasticity changes, as described by [100]. This form of expressing plasticity is relevant when several traits are analyzed together, so we can visualize which traits are more plastic than others. We calculated the *PPi* for each trait as the difference between the maximum and minimum mean values (max μ−min μ) divided by the maximum mean value:

(9) $$PPi=\frac{max\;\mu -min\;\mu }{max\;\mu }$$

### 4.11. Statistical Analyses

After checking the normality of the data, three-way analyses of variance (ANOVA) were performed in R v. 4.3.2 (R Development Core Team 2024) using ’Tgrowth’, ’light’, and ’water’ as fixed factors. In all cases, the data met the assumptions of normality and homoscedasticity; a significance level of *p* ≤ 0.05 was used, and model assumptions were checked. Statistically significant differences between groups were assessed using the HSD Tukey post hoc test in R v. 4.3.2 using the “agricolae” package. Principal Component Analysis (PCA) was performed in R version 4.3.2, utilizing the “stats” package for matrix calculations and the “factoextra” package for visualization support. The PCA was based on the correlation matrix, and the eigenvectors generated were analyzed to identify variables exhibiting a strong association with specific principal components (PCs). Pearson correlation coefficients were estimated using the “rcorr” function in R. Results are presented as means ± standard error (SE).

## 5. Conclusions

Our findings underscore the significant impact of elevated temperature on plant growth, biomass partitioning, and physiology of European beech seedlings. Specifically, we observed that warming decreased overall plant plasticity, reduced leaf size, allocated higher biomass to leaves, reduced leaf shedding, and increased metabolic and water demands, suggesting that global warming could constrain the adaptive capacity of beech regeneration, especially near the drier margins of its distribution. Warming reduced biomass due to a lower total leaf area and net photosynthesis but increased respiratory costs, diminished the potential for beech seedlings to perform the osmotic adjustment, and decreased the leaf safety margin, which may compromise the future survival of the species under dry conditions. Nevertheless, beech growth under shade might increase due to more and less costly leaves (lower *LMA*), which enhance transpiration rate and help cool down the leaf if warming occurs with deep soil and wet atmospheric conditions. This highlights the importance of understanding species-specific responses to multifactorial stress factors, which is essential for projecting the long-term persistence and ecological success of forest species under future warming scenarios.

## Figures and Tables

**Figure A4:**
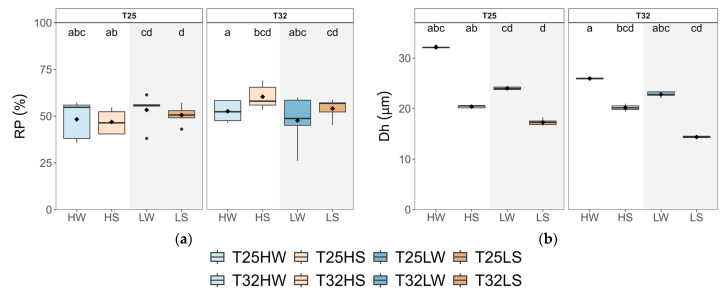
Boxplots of xylem morphological traits of beech seedlings grown in a factorial combination of temperature: 25 °C (T25) vs. 32 °C (T32), soil water availability: well-watered (W) vs. water-stressed (S), and light intensity: high (H) vs. low (L): (**a**) Xylem area occupied by radial parenchyma (Radial parenchyma, %); (**b**) hydraulic diameter (Dh, μm). The straight horizontal black line within each boxplot indicates the median, while the mean value is represented as a solid black point. Different letters indicate significant differences among treatments.

**Figure A5:**
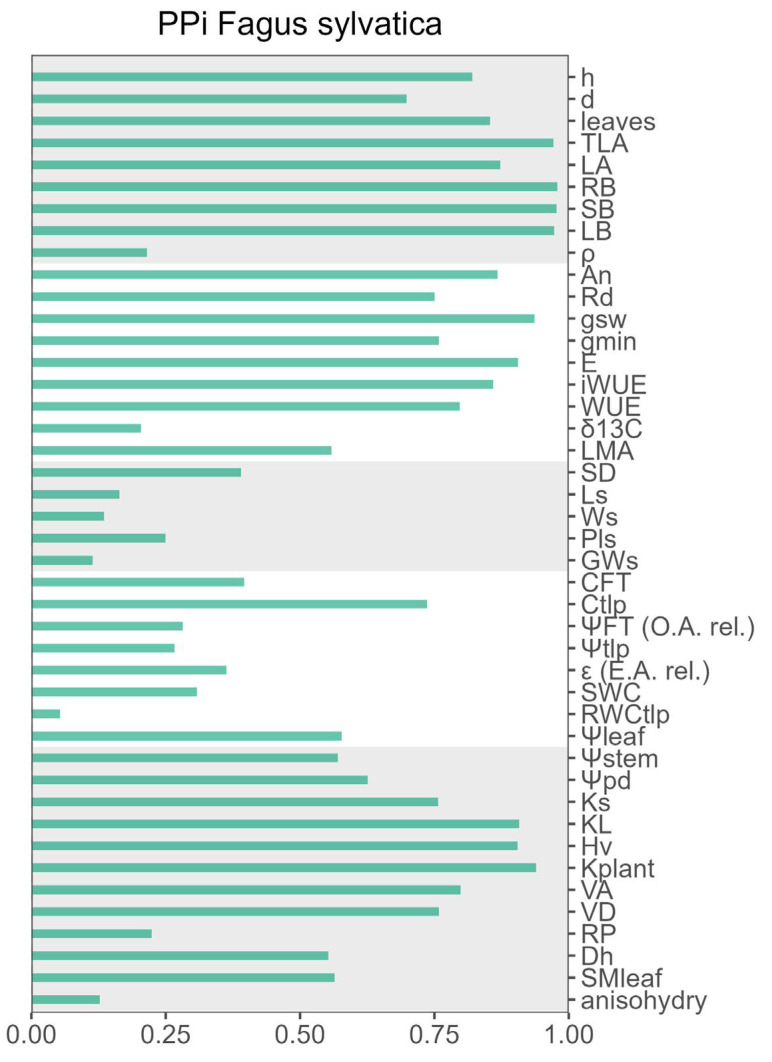
Relative response, or plasticity score, of each trait to the effect of each growing factor according to the Phenotypic Plasticity Index (PPI) as described by [100]. Gray and white shading highlights the different *F. sylvatica* organization levels ().

## Data Availability

All data supporting reported results can be found in the main text and in the Appendix A.

## Abbreviations

Abbreviations in this manuscript are shown in Table A1.

## Appendix A

**Table A1:** List of studied traits, with abbreviations and units and the classification to calculate the Phenotypic Plasticity index (PPi).

Symbol	Level for PPi	Definition	Values Units
h	Plant	Plant height	(cm)
d	Plant	Plant diameter	(mm)
leaves	Plant	Number of fully developed leaves	(nº)
TLA	Plant	Total plant leaf area	(cm^2^)
LA	Leaf GE relations	Average leaf area	(cm^2^)
RB	Plant	Root biomass	(g)
SB	Plant	Shoot biomass	(g)
LB	Plant	Leaf biomass	(g)
ρ	Plant	Wood density	(g cm^−3^)
A_n_	Leaf GE relations	Leaf net assimilation rate	(μmol CO_2_ m^−2^ s^−1^)
R_d_	Leaf GE relations	Leaf respiration	(μmol CO_2_ m^−2^ s^−1^)
g_sw_	Leaf GE relations	Leaf stomatal conductance to water vapor	(mol H_2_O m^−2^ s^−1^)
g_min_	Leaf GE relations	Leaf minimum conductance to water vapor	(mmol H_2_O m^−2^ s^−1^)
E	Leaf GE relations	Leaf transpiration	(mol H_2_O m^−2^ s^−1^)
iWUE	Not used for PPi	Intrinsic water use efficiency	(μmol CO_2_ mol^−1^ H_2_O)
WUE	Not used for PPi	Water use efficiency	(μmol CO_2_ mol^−1^ H_2_O)
δ^13^C	Not used for PPi	Carbon isotope composition	(‰)
LMA	Leaf GE relations	Leaf mass per area	(g m^−2^)
S_D_	Stomatal anatomy	Stomatal density	(nº stomata per mm^2^)
L_s_	Stomatal anatomy	Stomatal length	(μm)
PCI	Stomatal anatomy	Potential conductance index	(μm^2^ mm^−2^ 10^−4^)
W_s_	Stomatal anatomy	Stomatal complex width	(μm)
Pl_s_	Stomatal anatomy	Stomatal pore length	(μm)
GW_s_	Stomatal anatomy	Guard cell width	(μm)
C_Π100_	Leaf water relations	Leaf capacitance at full turgor	(MPa^−1^)
C_Π0_	Leaf water relations	Leaf capacitance at turgor loss point	(MPa^−1^)
Π^100^	Leaf water relations	Leaf osmotic potential at full turgor	(MPa)
Π^0^	Leaf water relations	Leaf turgor loss point	(MPa)
ε_leaf_	Leaf water relations	Leaf maximum Young’s modulus of elasticity	(MPa)
RWC _Π0_	Leaf water relations	Relative water content at the turgor loss point	(%)
SWC	Leaf water relations	Leaf-saturated water content	(g g^−1^)
Ψ_leaf_	Leaf water relations	Leaf midday water potential	(MPa)
Ψ_stem_	Leaf water relations	Stem midday water potential	(MPa)
Ψ_pd_	Not used for PPi	Predawn water potential	(MPa)
SM_leaf_	Not used for PPi	Leaf safety margin	(MPa)
Anisohydry	Plant	Ratio Ψ_leaf_ to Ψ_pd_	(dimensionless)
K_S_	Stem	Hydraulic specific conductivity	(kg m^−1^ s^−1^ Mpa^−1^)
K_L_	Stem	Leaf hydraulic conductivity	(mmol m^−1^ s^−1^ Mpa^−1^)
H_v_	Plant	Huber value	(m^2^ m^−2^)
k_plant_	Plant	Plant hydraulic conductance	(mmol m^−2^ s^−1^ Mpa^−1^)
V_A_	Stem	Average vessel lumen area	(μm^2^)
V_D_	Stem	Vessel density	(nº vessels per mm^2^)
RP	Stem	Xylem area occupied by radial parenchyma	(%)
D_h_	Not used for PPi	Hydraulic diameter	(μm)

**Table A2:** F-statistics from a full-factorial mixed effect ANOVA using growing temperature (Tgrowth), watering (Water) and light intensity (Light) as fixed effects with n = 5 replicates as random effect. Significant outcomes are indicated in bold with *p*-values < 0.05 indicated as *, *p* < 0.01 as ** and *p* < 0.001 as *** in the table. The factor or combination of growing factors with highest F score for each variable were marked in gray. Same abbreviations for variables than in Table A1.

Variables	Tgrowth	Light	Water	Tgrowth × Light	Tgrowth × Water	Light × Water	Tgrowth × Light × Water
h	**6.70 ***	**297.50 *****	**193.51 *****	3.16	0.76	**67.01 *****	<0.01
d	**42.33 *****	**520.49 *****	**98.20 *****	**42.15 *****	1.05	**29.80 *****	**6.72 ***
leaves	**100.86 *****	**145.62 *****	**188.08 *****	**7.40 ****	**23.78 *****	**34.68 *****	2.88
TLA	**9.40 ****	**300.24 *****	**276.75 *****	**39.46 *****	**45.09 *****	**150.84 *****	**60.06 *****
LA	**14.39 *****	**89.00 *****	**74.64 *****	**12.26 ****	0.89	**26.41 *****	1.16
RB (abs)	**41.51 *****	**240.44 *****	**149.30 *****	**45.23 *****	**24.45 *****	**109.47 *****	**35.29 *****
SB (abs)	**19.35 *****	**145.18 *****	**74.65 *****	**15.00 *****	**5.46 ***	**55.17 *****	**6.45 ***
LB (abs)	**8.48 ****	**267.33 *****	**122.04 *****	**15.91 *****	**18.65 *****	**90.60 *****	**27.50 *****
RB (rel)	0.30	0.71	**9.25 ****	**4.69 ***	**5.96 ***	0.02	**5.29 ***
SB (rel)	**15.35 *****	**4.59 ***	1.84	3.93	0.03	<0.01	**4.80 ***
LB (rel)	**25.41 *****	1.53	2.84	1.08	**4.37 ***	<0.01	1.17
A_n_	**18.99 *****	**169.38 *****	**52.01 *****	2.37	2.77	**7.88 ****	0.95
R_d_	**195.44 *****	**66.12 *****	**229.84 *****	3.53	0.19	**67.24 *****	**10.88 ****
g_sw_	0.01	**26.59 *****	**119.13 *****	1.21	**9.21 ****	**4.94 ***	0.70
g_min_	**652.60 *****	**700.90 *****	**1149.90 *****	**232.70 *****	**379.90 *****	**470.70 *****	**432.10 *****
E	0.15	**50.60 *****	**195.30 *****	2.74	**26.13 *****	2.79	2.03
iWUE	1.02	**108.38 *****	**390.07 *****	**77.93 *****	**17.56 *****	**18.80 *****	**84.06 *****
WUE	**7.26 ***	**59.41 *****	**97.25 *****	**38.82 *****	2.68	**6.81 ***	**44.05 *****
LMA	**264.22 *****	**505.64 *****	**166.07 *****	**22.42 *****	**111.74 *****	**7.13 ****	**118.75 *****
δ^13^C	**31.65 *****	**50.34 *****	**11.99 ****	0.08	0.37	0.13	0.32
S_D_	0.59	**10.49 ****	0.29	**4.55 ***	0.77	0.62	0.82
L_S_	<0.01	2.98	**11.35 ****	0.67	**6.55 ***	0.33	0.22
PCI	1.00	**27.23 *****	**6.87 ***	**7.18 ***	**11.39 ****	0.03	2.03
C_Π0_	1.49	**5.69 ***	**21.60 *****	1.00	**5.47 ***	3.01	0.22
C_Π100_	**33.79 *****	**22.46 *****	**86.54 *****	0.49	**14.93 *****	**22.86 *****	0.33
Π^0^	**5.02 ***	1.43	2.46	0.11	**11.08 ****	**6.28 ***	1.67
Π^100^	0.27	**4.25 ***	**16.44 *****	1.40	**16.27 *****	**4.50 ***	0.88
ε_leaf_	0.28	0.04	**9.40 ****	0.11	**7.08 ***	**4.19 ***	0.66
Ψ_leaf_	**69.36 *****	**29.73 *****	**737.58 *****	**6.85 ***	0.01	<0.01	**4.305 ***
Ψ_stem_	**59.97 *****	**15.49 *****	**710.44 *****	**12.86 ****	0.07	0.35	0.75
Ψ_pd_	**46.04 *****	**11.67 ****	**852.71 *****	1.87	0.03	3.55	4.87 *
SM_leaf_	**13.28 *****	3.69	**107.36 *****	0.41	**5.42 ***	**5.53 ***	**29.57 *****
K_s_	0.14	**32.26 *****	**28.46 *****	0.38	0.81	0.01	0.93
K_l_	**90.41 *****	0.40	**221.90 *****	0.85	**74.61 *****	**27.33 *****	**54.54 *****
Hv	**76.66 *****	**15.01 *****	**99.57 *****	1.45	**55.79 *****	1.05	**6.37 ***
k_plant_	0.39	**23.98 *****	**256.35 *****	0.01	**7.94 ****	2.54	3.85
V_D_	**48.24 *****	**447.68 *****	**2158.38 *****	**55.35 *****	**42.96 *****	**5.88 ***	**87.85 *****
V_A_	**261.49 *****	**893.15 *****	**2680.29 *****	**90.51 *****	**90.14 *****	**111.22 *****	**148.31 *****
RP	2.17	0.05	0.93	3.67	3.00	0.06	<0.01
D_h_	**245.32 *****	**1165.82 *****	**3405.51 *****	**11.11 ****	**52.28 *****	**14.03 *****	**190.31 *****

**Table A3:** Phenotypic plasticity index: (max mean–min mean)/max mean temperature response to light for each group of variables. Bold numbers represent significant differences between light treatments.

Group of Variables	Measured Variables	Well-Watered		Water Deficit	
		T25	T32	T25	T32
		Response to Light (HW vs. LW)	Response to Light (HW vs. LW)	Response to Light (HS vs. LS)	Response to Light (HS vs. LS)
Plant level	10	**0.67**	0.52	0.50	**0.56**
Stem	6	**0.40**	0.18	**0.29**	**0.26**
Leaf (PV + GE)	13	**0.32**	**0.32**	**0.30**	0.21
Leaf hydric relations	6	**0.19**	**0.18**	0.08	**0.11**
Leaf GE relations	7	**0.43**	**0.44**	**0.49**	0.29
Overall	29	**0.46**	0.35	**0.37**	**0.35**

**Table A4:** Phenotypic plasticity index: (max mean–min mean)/max mean temperature response to water for each group of variables. Bold numbers represent significant differences between watering treatments.

Group of Variables	Measured Variables	High Light		Low Light	
		T25	T32	T25	T32
		Response to Water (HW vs. HS)	Response to Water (HW vs. HS)	Response to Water (LW vs. LS)	Response to Water (LW vs. LS)
Full plant	10	**0.67**	0.57	Full plant	10
Stem	7	**0.51**	0.32	Stem	7
Leaf (PV + GE)	12	0.37	**0.41**	Leaf (PV + GE)	12
Leaf water relations	6	**0.33**	0.16	Leaf water relations	6
Leaf GE relations	6	0.39	**0.61**	Leaf GE relations	6
Overall	29	**0.50**	0.44	Overall	29

**Table A5:** Summary table of all the measured parameters, expressed as mean ± standard error. Sample size is referred as n. Variables are described in Table A1.

Growth Temperature		T25				T32			
Treatment		HW	HS	LW	LS	HW	HS	LW	LS
Sample Size (n)		6	6	6	6	6	6	6	6
h	(cm)	61.9 ± 2.3	28.4 ± 1.6	18.9 ± 1.2	11.3 ± 0.5	63.1 ± 3.9	27.2 ± 0.8	26.2 ± 2.3	16.3 ± 1.6
d	(mm)	12.6 ± 0.2	9.0 ± 0.3	4.7 ± 0.5	4.6 ± 0.2	9.4 ± 0.4	6.6 ± 0.1	5.4 ± 0.3	3.8 ± 0.2
leaves	(nº)	23 ± 2	11 ± 1	10 ± 2	3 ± 0	41 ± 3	16 ± 1	20 ± 1	10 ± 0
TLA	(cm^2^)	1881.76 ± 113.43	214.56 ± 8.03	165.04 ± 54.89	31.34 ± 8.24	986.67 ± 68.17	328.22 ± 31.02	273.95 ± 26.37	86.74 ± 22.89
LA	(cm^2^)	36.16 ± 2.02	17.88 ± 1.71	13.37 ± 1.48	9.62 ± 0.42	25.41 ± 2.98	11.51 ± 1.1	13.65 ± 1.37	9.28 ± 0.71
RB	(g)	30.9 ± 3.1	8 ± 1.3	2.9 ± 0.5	1.4 ± 0.1	14.5 ± 0.8	4.6 ± 0.3	2.7 ± 0.6	0.7 ± 0.1
SB	(g)	16.3 ± 0.8	2.6 ± 0.6	0.5 ± 0	0.4 ± 0.1	8.6 ± 0.7	3.9 ± 0.4	1.4 ± 0.2	0.5 ± 0.1
LB	(g)	49.7 ± 4.1	8.7 ± 1.2	3 ± 0.5	1.5 ± 0.1	21.2 ± 2.2	5.6 ± 0.3	4.7 ± 0.6	1 ± 0.2
TB	(g)	95.8 ± 6.5	19.3 ± 2.5	6.6 ± 1.1	4.1 ± 0.8	46.9 ± 2.6	14.8 ± 1	9 ± 1.3	2.5 ± 0.5
RB	(%)	32.4 ± 2.6	38.6 ± 1.9	43.2 ± 4	41.8 ± 1.6	32.7 ± 3	30.8 ± 0.8	28.7 ± 2	34.9 ± 2.2
SB	(%)	15.9 ± 1.1	13.1 ± 0.3	10.7 ± 2	11.7 ± 2.5	17.1 ± 1.1	24.7 ± 1.2	18.1 ± 0.9	22.6 ± 3.8
LB	(%)	50 ± 1.3	44.9 ± 1	43.8 ± 1.4	46.5 ± 1.3	45.3 ± 4.1	40.3 ± 2	55 ± 1	42.4 ± 2.7
K_s_	(kg m^−1^ s^−1^ Mpa^−1^)	25.94 ± 4.21	14.78 ± 0.92	14.56 ± 0.91	6.11 ± 1.31	22.09 ± 2.58	16.85 ± 2.23	15.86 ± 1.93	7.19 ± 2.61
K_L_	(mmol m^−1^ s^−1^ Mpa^−1^)	0.62 ± 0.11	5.26 ± 0.15	1.72 ± 0.32	2.84 ± 0.45	0.81 ± 0.12	1.37 ± 0.07	0.44 ± 0.08	1.62 ± 0.23
K_plant_	(mmol m^−2^ s^−1^ Mpa^−1^)	2.46 ± 0.31	0.96 ± 0.13	1.92 ± 0.29	0.34 ± 0.02	2.93 ± 0.12	0.32 ± 0.01	1.96 ± 0.06	0.18 ± 0.03
H_V_	(cm^2^ m^−2^)	7.6 ± 1	59.8 ± 6.4	21.9 ± 3.8	64.5 ± 12.1	7.2 ± 0.8	9.9 ± 0.9	6 ± 0.7	26 ± 3.2
LMA	(g m^−2^)	82.9 ± 1.4	90.2 ± 2.1	62.2 ± 1.4	49.6 ± 1.5	80.1 ± 1.9	43.9 ± 1.1	49.5 ± 0.7	40.5 ± 0.7
A_n_	(μmol m-2 s-1)	8.4 ± 0.5	6.1 ± 0.7	3.4 ± 0.6	2.2 ± 0.2	7.4 ± 0.4	3.5 ± 0.1	2.7 ± 0.4	1.1 ± 0.4
R_l_	(μmol m^−2^ s^−1^)	0.75 ± 0.02	0.25 ± 0.01	0.41 ± 0.02	0.35 ± 0.02	1.00 ± 0.04	0.61 ± 0.04	0.72 ± 0.03	0.51 ± 0.03
g_sw_	(mmol m^−2^ s^−1^)	232.7 ± 46.7	101.9 ± 13	124.8 ± 24	33.2 ± 3	271.4 ± 14	31.3 ± 0.5	171 ± 6.8	17.2 ± 3.2
g_min_	(mmol m^−2^ s^−1^)	2.36 ± 0.06	1.75 ± 0.05	1.88 ± 0.02	1.35 ± 0.01	5.57 ± 0.08	1.78 ± 0.1	2.24 ± 0.01	1.81 ± 0.05
E	(mol m^−2^ s^−1^)	3.2 ± 0.4	1.8 ± 0.2	1.9 ± 0.3	0.6 ± 0	3.9 ± 0.1	0.7 ± 0	2.7 ± 0.1	0.4 ± 0.1
iWUE	(μmol mol^−1^)	38.8 ± 5.3	63.1 ± 0.9	25.4 ± 0.4	73.2 ± 4.7	27.8 ± 2.6	110.4 ± 1.7	14.7 ± 1.9	36.5 ± 5
WUE	(μmol mol^−1^)	2.7 ± 0.2	3.3 ± 0.1	1.8 ± 0	4 ± 0.3	1.9 ± 0.1	5.3 ± 0.4	1.1 ± 0.2	1.5 ± 0.2
δ^13^C	(‰)	−29.8 ± 0.5	−27.6 ± 0.7	−32.3 ± 0.7	−30.9 ± 0.8	−31.5 ± 0.4	−30.4 ± 0.7	−34.8 ± 0.3	−33.5 ± 0.6
